# Safer and faster: evaluation of a dental implant checklist

**DOI:** 10.1007/s00784-026-06986-6

**Published:** 2026-06-29

**Authors:** Johannes Raphael Kupka, Franck Renouard, Keyvan Sagheb, Bilal Al-Nawas, Alexander W. Eckert, Eik Schiegnitz

**Affiliations:** 1 Department of Oral and Maxillofacial Surgery, Plastic Surgery, University Medical Center of the Johannes Gutenberg-University, Augustusplatz 2, Mainz, 55131 Germany; 2 Department of Oral and Maxillofacial Surgery, University Hospital of the Paracelsus Medical Private University, Nürnberg, Germany; 3 Private Practice Oral Surgery Paris, Paris, France

**Keywords:** Dental implant, Surgical safety checklist, Human factors, Clinical deviations, Workflow disruptions, Efficiency

## Abstract

**Objective:**

Systematic safety approaches, remain largely unevaluated in dental implantology. This prospective interventional study aimed to evaluate the impact of a structured surgical safety checklist on clinical incidents and organizational efficiency in dental implant surgery.

**Methods:**

A before-and-after study design was implemented at a high-volume center, documenting 124 consecutive dental implant surgeries (61 without, 63 with checklist) using a previously by Kupka et al. developed safety checklist. The primary endpoint was the occurrence of incidents divided into clinical deviations and workflow disruptions; the secondary endpoint was procedure duration.

**Results:**

Checklist implementation resulted in a statistically significant reduction in mean surgery duration. Mean operative time decreased from 75.4 min (SD 31.9) to 60.3 min (SD 31.4), representing a median decrease: 15.0 min (*p* < 0.01). The rate of surgeries with at least one incident decreased from 36.1% (22/61) to 23.8% (15/63), which was not statistically significant (*p* = 0.170). However, clinical deviations dropped significantly from 13.1% to 0.0% (*p* = 0.003).

**Conclusion:**

To our knowledge, this is the first study investigating checklist effects on incidents and operating time in implant dentistry, and the largest case number in oral surgery. Introducing a surgical checklist significantly improved efficiency by reducing procedure duration. While the reduction in overall incidents was not statistically significant, the elimination of clinical deviations suggests a safety benefit. Structured checklists might be a valuable tool to enhance patient safety and operational workflow in elective implant surgery.

**Supplementary Information:**

The online version contains supplementary material available at 10.1007/s00784-026-06986-6.

## Introduction

Unforeseen events and human errors are among the most frequent causes of complications in healthcare systems, with up to 80% of errors being of non-technical origin [1]. Many preventive techniques from the field of human factors research have already been adopted from aviation into medical practice, with anaesthesiology being a pioneering discipline [2]. The use of checklists also originated in aviation, where they have served for decades as a central tool to ensure safety and efficiency. Their purpose is not only to act as a cognitive aid but also to enhance team communication [3].

Although the knowledge required to prevent errors often exists among clinicians, errors still occur [4]. Checklists have therefore proven remarkably effective in preventing so-called “never events,” such as wrong-site surgery. A retrospective analysis showed that 21.1% of all wrong-side errors could be avoided through the use of a checklist [5].

With the introduction of the World Health Organization (WHO) Surgical Safety Checklist, a structured instrument was established for the first time to ensure the quality of surgical procedures independently of individual experience [6]. Large international studies have demonstrated that the use of such checklists not only reduces postoperative morbidity and mortality but also significantly improves team communication, standardized preparation, and overall patient safety [7]. Several authors have confirmed these findings in more specific contexts. For example, McCarroll et al. reported a 36% reduction in postoperative complications and a 62% reduction in mortality [8]. Additional success stories are summarized in several review articles [9–11]. The relevance of these tools has also been recognized in clinical practice; for instance, the American Congress of Obstetricians and Gynecologists (ACOG) strongly supports the use of protocols and checklists to improve patient care and standardize practices [8].

Despite this well-established evidence and the rapid growth and increasing complexity of implant dentistry, data for oral surgery—and particularly for implantology—remain limited [9, 12]. Systematic safety approaches have not yet been widely integrated or evaluated. The relative scarcity of evidence in implantology likely reflects the unique procedural and organizational structure of dental surgery. Implant procedures are often performed in small, independent practices rather than large hospital systems, making the systematic implementation and evaluation of safety protocols more challenging [13]. This structural setting may explain why safety protocols, despite their proven effectiveness in hospital environments, have not yet been routinely adopted in implant dentistry. It should also be noted that the importance of checklists is not taught in dental school. Furthermore, students are not required to use checklists during their university hospital training. Given the elective nature of implant surgery and the potential for long-term biological and mechanical complications, structured safety tools are of high relevance [13, 14]. Nevertheless, empirical data quantifying their benefits in implant surgery are still lacking.

The introduction of a standardized checklist aims to reduce organizational incidents and optimize surgical duration. Therefore, this study was conducted in a high-volume implantology center to systematically evaluate the implementation of such a checklist in dental implantology. Specifically, we assessed its impact on complication rates, surgical duration, and thereby organizational efficiency.

We hypothesize that the introduction of a structured checklist will significantly reduce incidents during implant surgery while improving workflow efficiency.

## Materials and methods

### Ethics

The study was reviewed and approved by the Ethics Committee of Rhineland-Palatinate (Approval No. 2022–16629). As no patient-identifiable data were collected, individual consent was waived by the ethics committee.

### Study design

This was a prospective interventional quality assessment and improvement study. The study was conducted at a single center (University Medical Center Mainz) and all participating surgeons were experienced board-certified specialists with a minimum of 10 years of practice. Surgery was carried out with at least one assistant. A before-and-after study design was used, comparing outcomes prior to and following checklist implementation over one and a half years. This study was structured and is reported in accordance with the SQUIRE 2.0 (Standards for QUality Improvement Reporting Excellence) guidelines for quality improvement initiatives, incorporating relevant elements of the STROBE (Strengthening the Reporting of Observational Studies in Epidemiology) statement for its observational before-and-after design. The completed STROBE checklist has been provided as supplementary material (Supplementary file 2). Because this initiative was conducted as an internal quality assessment and improvement project focusing primarily on clinical workflow efficiency and institutional complication tracking using fully anonymized data, prospective public clinical trial registration was not initiated.

### Checklist development

The surgical safety checklist used in this study had previously been developed and published by Kupka et al. as part of a scoping review [9]. It was based both on the available literature and on the clinical experience of practitioners at the University Medical Center Mainz. The scoping review included publications by Remiszewski, Bidra, and Christman [15–17]. The purpose of the checklist was to ensure that all safety aspects of dental implant surgery were considered and to promote consistency and accountability in the pre- and postoperative management of patients.

The checklist was structured into three sections, corresponding to the typical course of implant placement: planning and information, preoperative, and postoperative. Data from the UK National Patient Safety Agency indicate that the majority of preventable safety incidents in dentistry occur in the pre- or postoperative phases. For this reason, the intraoperative period was not included.

Following the recommendations of Renouard et al. and Gawande, the checklist was deliberately limited to fewer than ten items to ensure clarity and efficient use. Each item could explicitly be answered with “yes” or “no,” providing transparency as to whether a point had been consciously negated or inadvertently overlooked.

### Implementation

The checklist was introduced in the Department of Oral and Maxillofacial Surgery at the University Medical Center Mainz. As an initial step, incidents during implant surgeries were documented using a standardized form to establish a baseline for the subsequent quality assessment study. It consisted of predefined items that could be marked as present or absent, and an optional free-text field was available for additional events (Supplementary file 2). The items included: patient informed > 24 h before surgery, correct patient, correct implant position, allergic reaction, abnormal blood glucose, unexpected bleeding, intraoperative pain, nerve injury, damage to adjacent teeth, delays due to missing/incorrect materials or documents, missing radiographs, missing premedication, and other incidents noted in free text. The surgeon also recorded whether the incident was quickly managed or required assistance from another practitioner. The study supervisor ensured that all data was entered immediately after each procedure. After more than 60 procedures had been documented, the checklist was formally implemented.

Participating surgeons were personally informed about the introduction of the checklist and instructed in its use. It was implemented as a paper-based form, placed next to the documentation computer for immediate accessibility during each procedure. Background information about the checklist was made freely available online. The study supervisor ensured that the checklist was actually used. For each surgery, incidents were recorded, along with the duration of the procedure, immediate implant placements, the performance of guided bone regeneration (GBR), internal/external sinus floor elevations and the number of implants placed.

### Study population and selection criteria

Patients undergoing dental implant surgeries were enrolled. The inclusion criteria were broad: any patient scheduled for dental implant placement under local anesthesia was eligible, with no restrictions regarding age or biological sex. Surgeries performed under general anesthesia were excluded from the study.

To check for comparability between the pre-checklist and post-checklist groups, patient demographics (age, sex) and key procedural complexity drivers—specifically bone augmentation procedures (including internal and external sinus lifts) and immediate implant placement—were recorded and analyzed.

### Study endpoints

The primary endpoint was the occurrence of incidents during implant surgery. These were divided into clinical deviations and workflow disruptions for a more detailed analysis. The secondary endpoints were procedure duration and the number of implants placed per surgery.

### Statistical power and sample size calculation

An a priori sample size calculation was performed based on the expected reduction of specific workflow disruptions (missing/malfunctioning materials or instruments). Assuming a disruption rate of 22.5% in the non-checklist group and a reduction to 5.0% in the checklist group [18], a sample size of 108 surgeries (54 per group) was required to achieve a statistical power of 80.8% with a one-tailed significance level of 0.05.

To account for potential data loss, a target sample size of *n* = 114 was initially planned. Data collection followed a convenience sampling strategy, where surgeries were included based on the presence and availability of the principal investigator to oversee checklist implementation. Ultimately, a total of 124 surgeries (61 without and 63 with the checklist) were successfully included, meeting and exceeding the required sample size.

### Data collection and statistical analysis

All data were entered into standardized spreadsheets (Excel; Microsoft Corp., Redmond, WA, USA). Descriptive statistics were calculated. Data distribution was assessed using the Shapiro–Wilk test. Comparisons between the pre-implementation and post-implementation periods were conducted. As normality was not confirmed, the Mann–Whitney U test was used for surgical duration and number of implants, while students t-test could be used for patient age. Categorical outcomes were compared using Fisher’s exact test. For the primary endpoints, effect sizes were expressed as relative risks (RR) with their corresponding 95% confidence intervals (CI). In cases where an event rate of 0% precluded the calculation of the relative risk, the Risk Difference (RD) with its 95% CI was reported instead. Statistical analyses were conducted using jamovi (Version 2.7.31; The jamovi Project, Sydney, Australia), an R-based software platform. All p-values were two-sided, and a significance level of 0.05 was applied.

## Results

**Fig. 1:**
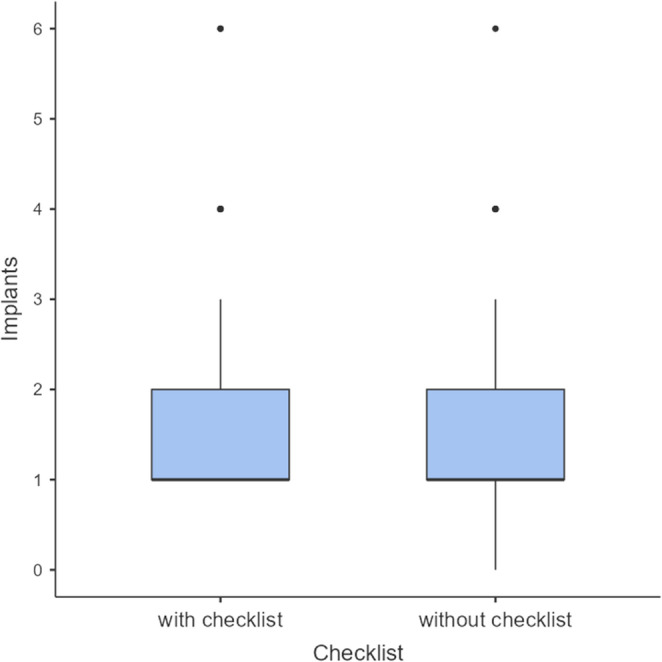
Distribution of the number of implants placed per surgery before (*n* = 61) and after checklist implementation (*n* = 63). No significant difference was observed between groups. Boxes represent the interquartile range, the line inside the box indicates the median

A total of 124 dental implant surgeries were documented during the study period, comprising 61 procedures before checklist implementation and 63 with the checklist. Across all surgeries, 227 implants were placed. The average number of implants placed per procedure was 1.83 (SD 1.19), with no significant difference between groups (without checklist 1.80, SD 1.23 vs. with checklist 1.86, SD 1.15; *p* = 0.904, Mann–Whitney U test)(Fig. 1).

Further study group characteristics are mentioned below in Table 1.

**Table 1:** Baseline demographics and clinical characteristics of the study population. Data are presented as absolute numbers (percentages) unless stated otherwise

	Without checklist (*n* = 61)	With checklist (*n* = 63)	*p*
Bone augmentation (GBR)	17 (27.9%)	19 (30.2%)	0.844^1^
Internal sinus lift	3 (4.9%)	1 (1.6%)	0.361^1^
External sinus lift	3 (4.9%)	4 (6.3%)	1.000^1^
Immediate implant	4 (6.6%)	6 (9.5%)	0.744^1^
Sex (female)	33 (54.1%)	27 (42.9%)	0.281^1^
Age (years), Mean (± SD)	59.3 (± 13.4)	62.7 (± 11.8)	0.130^2^
Implants per surgery (± SD)	1.80 (± 1.23)	1.86, (± 1.15)	0.904^3^

*SD* standard deviation, *GBR* guided bone regeneration. Data for bone augmentation (GBR) were collected independently from sinus floor elevations; thus, the GBR group does not contain internal or external sinus lift cases

^1^Calculated using Fisher's exact test; ^2^Calculated using Student's t-test; ^3^Calculated using Mann–Whitney U test

**Fig. 2:**
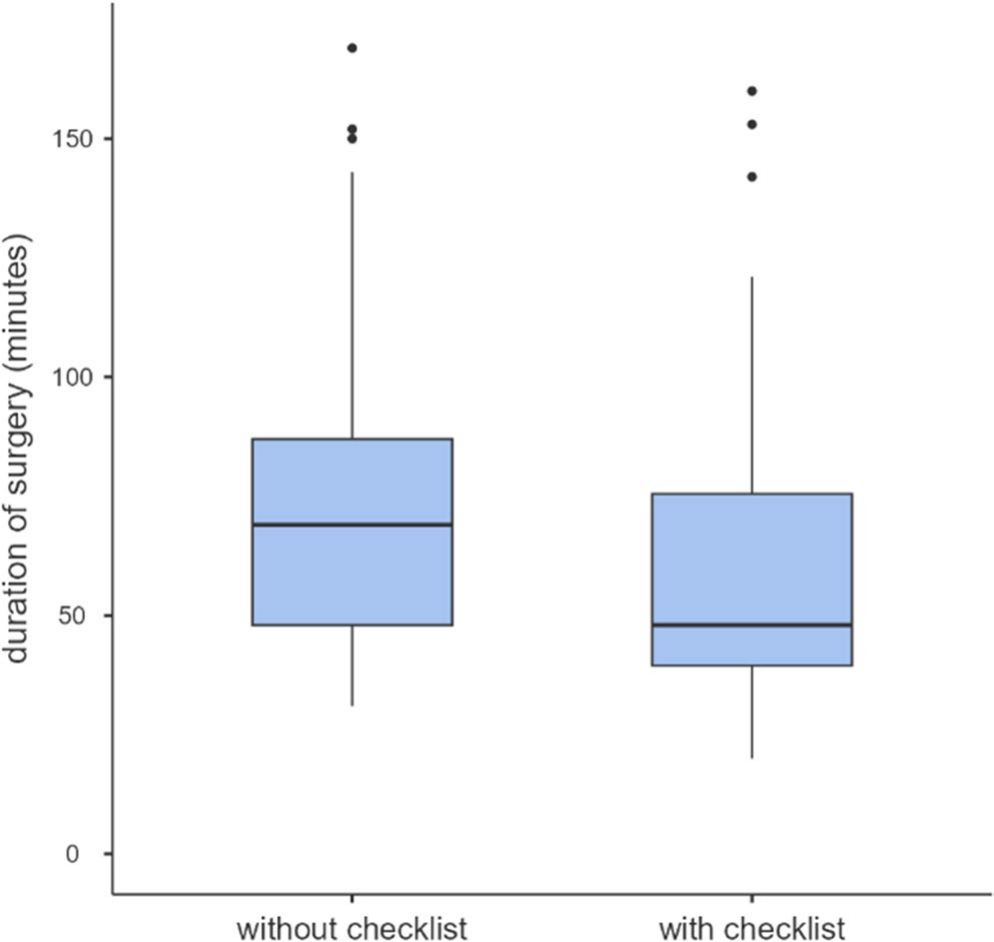
Duration of dental implant surgeries before (*n* = 61) and after checklist implementation (*n* = 63). Surgeries performed with the checklist were significantly shorter (mean difference: 15.0 min; Mann–Whitney U test, *p* < 0.01). Boxes represent the interquartile range, the line inside the box indicates the median

The mean duration of surgery was 67.3 min overall. Before checklist use it was 75.4 min (SD 31.8, median 69, IQR 48–87), and after implementation 60.3 min (SD 31.4, median 48, IQR 39.5–75.5), showing a statistically significant reduction (*p* < 0.01, Mann–Whitney U test) with a moderate effect size with a rank-biserial correlation of −0.320. The mean difference was 15.0 (95% CI: 5.0–24.0 min) (Fig. 2).

In surgeries performed without the checklist, 33 incidents were documented, occurring in 22 operations (36.1%). That included 8 clinical deviations in 8 surgeries (13.1%) and 25 workflow disruptions in 19 surgeries (31.1%). The most frequent issue was missing material (*n* = 14). Further workflow disruptions included missed antibiotic intake (*n* = 2), defective material (*n* = 1), the need for assistance from another surgeon (*n* = 1), missing replacement after instrument fracture (*n* = 1), impaired implant placement due to prosthetic restorations (*n* = 1), missed premedication (*n* = 1), missing radiographs (*n* = 3), and missing documents (*n* = 1). Clinical deviations included wrong sectioning of a bridge (*n* = 1), injury of neighboring structures (*n* = 3), abnormal blood glucose level (*n* = 1), wound dehiscence (*n* = 1), incorrect implant position (*n* = 1), patient pain due to substance abuse with insufficient midazolam effect (*n* = 1).

In surgeries performed with the checklist, 16 workflow disruptions were observed in 15 surgeries (23.8%). These included missing material (*n* = 9), missed antibiotic intake (*n* = 1), missed premedication (*n* = 1), missing radiographs (*n* = 2), and missing documents (*n* = 3).

The statistical comparison of surgeries with at least one incident between the two periods revealed no significant difference (*p* = 0.170, Fisher’s exact test; RR = 0.66, 95% CI: 0.379–1.15). However, a highly significant impact was found when analyzing the specific subtypes of incidents. Clinical deviations dropped from 13.1% (8/61 surgeries) in the group without the checklist to 0.0% (0/63 surgeries) after its implementation (*p* = 0.003, Fisher’s exact test). Due to the zero-event rate in the post-checklist cohort, the relative risk was not calculable; therefore, the effect size is reported as Risk Difference (RD = −13.1%, 95% CI: −21.6% to −4.6%).

In contrast, the occurrence of workflow disruptions showed no statistically significant change, with 31.1% of surgeries (19/61) affected before versus 23.8% (15/63) after checklist implementation (*p* = 0.423, Fisher’s exact test; RR = 0.827, 95% CI: 0.541–1.27).

## Discussion

Dental implant surgery is unique in that it is an elective procedure [14]. Consequently, there is a strong clinical rationale for implementing a safety checklist to standardize workflows. The demands on planning and precision are particularly high, as the long-term success of an implant often depends on the relatively short intraoperative phase [19]. Errors occurring during surgery are frequently difficult to reverse and can lead to biological as well as mechanical complications [13, 20]. Considering that patient compliance represents an uncontrollable but highly relevant prognostic factor, all controllable factors should be optimized [21].

Furthermore, dental implants are not covered by public health insurance in most countries, and thus represent a substantial private expense for patients, while also being economically relevant for practitioners [22, 23]. Both perspectives, however, lose significance if complications, re-interventions, or excessive delays occur.

To our knowledge, this is the first study in implant dentistry to investigate the impact of a structured checklist on clinical incidents, workflow disruptions, and operating time [9, 12]. Within oral and maxillofacial surgery, it also represents the study with the largest case number to date [12]. Schmitt et al. previously conducted a pioneering investigation involving 40 surgeries per group and demonstrated both a reduction in complications and a high level of staff satisfaction with improved communication within the team and reduce stress level. Their results already suggested that checklists are well accepted by surgical teams and improve safety outcomes [18].

Other publications, such as those by Saksena and Liew, reported extended time periods (30 and 18 months, respectively) without complications, yet did not provide exact case numbers [24, 25]. Moreover, Saksena’s focus was on the extraction of wrong teeth—a rare but severe error, which is difficult to measure and prone to selection bias [25]. In contrast, implant surgery demands a higher level of procedural standardization.

In our study, the two groups demonstrated comparable baseline characteristics, with no statistically significant differences regarding patient age, sex, or parameters of surgical complexity—including the number of implants placed, immediate implant placements, and the performance of guided bone regeneration (GBR) or internal/external sinus floor elevations. Interestingly, the checklist group even showed slightly higher descriptive values for implant numbers, immediate implant placements, performance of guided bone regeneration (GBR) and external sinus floor elevations supporting that the significant reduction in clinical deviations and surgery duration was not biased by simpler case selections in the second phase. A before-and-after design was chosen, as randomization could have introduced learning effects during the study. Similar designs have been used in other large-scale investigations, such as the seminal study by Haynes et al.[26, 27]. Since all surgeries in this study were performed exclusively by highly experienced clinicians, a relevant confounding effect through the acquisition of new surgical skills during the study period is unlikely. However, this non-randomized approach cannot completely rule out time-related effects or a temporary increase in staff awareness (Hawthorne effect) following the introduction of the new protocol. Our findings indicate that while the descriptive reduction in overall incidents did not reach statistical significance, the checklist might have a significant impact on preventing clinical deviations.

In implant dentistry, complication rates are generally low, and implants often remain in situ for years without issues [20]. Complications tend to occur intraoperatively or in the early postoperative phase—often due to workflow disruptions, such as missing materials [9, 13]. Consequently, our study tracked both organizational workflow disruptions and clinical deviations [28]. While workflow disruptions remained statistically unchanged, clinical deviations—such as minor soft-tissue injuries or incorrect implant positioning—were rare overall and completely absent in the checklist phase.

Our checklist was designed to take less than one minute to complete—an aspect previously highlighted as crucial for sustained compliance[29]. Concerns about time loss are often unfounded: in other studies, such as that by Kearns et al., the perceived inappropriateness of checklists for certain settings dropped after regular use [3]. On the contrary, literature suggests that checklist utilization is frequently associated with an enhanced perception of safety and preparedness among team members. Schmitt et al. reported that surgeons, assistants, and nurses felt better prepared when the checklist was used and even missed it when it was absent [18]. Similarly, Panesar et al. demonstrated high staff satisfaction in orthopedic surgery and anesthesiology, emphasizing improved communication and workflow following checklist implementation [5].

Few studies have examined the actual effect of checklists on procedure duration. McCarroll et al. observed no significant change in operating time, although a reduction in readmissions was noted [8]. In contrast, we found a significant median decrease of 15.0 min per surgery. Considering that the mean baseline duration was 75.4 min, this reduction can be clinically and operationally relevant. The time savings likely stem from avoiding delays such as material retrieval or additional consultations, and from improved intraoperative communication [30]. Checklists are known to enhance team communication and flatten hierarchical barriers, especially during the “time-out” phase. This contributes to smoother workflow and greater preparedness among all participants [31]. These factors offer a plausible explanation for the reduced operating time observed in our cohort.

Although such effects are challenging to quantify, previous studies have captured them through staff questionnaires [18]. The observed reduction in operating time in our cohort thus likely reflects a multifactorial benefit, combining better preparation, role clarity, and team communication. By ensuring that all team members are aware of the key patient factors and procedural requirements before surgery, the checklist facilitates a shared mental model and prevents unnecessary disruptions during the operation [32].

A reduction in operating time of approximately 15 min also carries potential economic implications. To provide an exploratory estimate based on these findings, a hypothetical cost-benefit calculation can be formulated. Even after accounting for the estimated two minutes required to complete the checklist, a net time gain of about 13 min per procedure remains. Assuming as a theoretical baseline that a German dental practice must generate approximately €204 per hour to cover operating costs, this time saving would translate to an assumed economic benefit of 44.2€ per procedure. When considering the average hourly revenue required to achieve profitability (approximately €334 per hour), the potential added value could theoretically increase to around 72.4€ per case. These speculative values are based on published average hourly rates for German dental practices [33]. Actual figures may vary depending on regional and structural practice factors. Furthermore, the actual financial benefit depends on how the saved time is utilized within each practice.

Beyond pure economic efficiency literature shows that the longer the duration of surgery, the greater the risk of error. For instance Cheng H, et al. (2018), “Prolonged operative duration is associated with complications: a systematic review and meta-analysis”[34]. This systematic meta-analysis of 66 studies observes that each additional 30 minutes of operating time increases the risk of postoperative complications by 14%. The risk of complications doubles when the operation exceeds 2 hours. Consequently, minimizing operative duration appears to be a critical factor in mitigating postoperative risks across various surgical specialties, further supporting the clinical utility of time-saving checklists.

Compliance with checklist utilization was not formally quantified in this study, although descriptive observations suggest that sustained adherence is heavily contingent on active supervision. As emphasized by Kearns et al., the presence of dedicated “champions” or human factors officers is critical to maintaining adherence [3]. Remiszewski et al. previously published data on checklist compliance, reporting an adherence rate of 100%. Although the authors noted that completing the checklist required additional time, this did not appear to affect compliance [15]. It should be noted, however, that the participants in that study were residents still in training. This context may have contributed to the exceptionally high compliance rate, as structured workflows are often more strictly followed in academic or supervised settings compared with private practice environments.

Accumulating evidence from human factors research demonstrates that structured training significantly contributes to mitigating adverse events. Overall, teamwork and communication training have been shown to enhance the safety culture in clinical settings, which directly correlates with optimized patient outcomes [35]. Longitudinal evidence indicates that such training only achieves significant improvements in patient safety culture and teamwork after approximately 12 months of sustained application. Importantly, these improvements are not limited to non-technical skills; enhanced team performance, particularly in the domain of mutual support, has been directly associated with higher departmental patient safety grades, suggesting a tangible transfer into better clinical outcomes [36].

In our study, all surgeries were performed by highly experienced surgeons. The observed elimination of clinical deviations alongside the significant decrease in operative duration thus highlights the practical effectiveness of checklists, even in a high-expertise environment. The medical literature shows that clinicians who perform procedures routinely and frequently (high-volume clinicians) generally experience fewer complications than lower-volume counterparts [37–39]. Nevertheless, our findings indicate that structured safety tools provide an additional clinical and organizational benefit that extends beyond clinical seniority alone. But targeted application of these tools is crucial. Duplication of existing safety measures can reduce adherence, and each clinician needs to identify and consistently apply the most suitable instruments for their practice [40]. The thoroughness of the surgical team is central to the effectiveness of checklists, and routine without reflection can undermine their intended benefit [18].

Several limitations of this investigation must be explicitly acknowledged. First, the non-randomized, single-center, before-and-after study design introduces a theoretical risk of a Hawthorne effect and potential case-mix imbalances over time. Consequently, time-related confounding or subtle shifts in institutional routine cannot be entirely ruled out, preventing the deduction of a direct causal effect. Nevertheless, this remains the most practical and established approach for clinical interventions involving behavioral adaptation [41].

Second, because this study focused exclusively on highly experienced, high-volume clinicians to minimize learning curves, the findings may not be directly generalizable to training environments, less experienced surgeons, or standard private dental practices with lower surgical volumes where workflows differ significantly.

Third, organic compliance with the checklist could not be measured because its implementation was consistently monitored and supervised by the principal investigator. While this rigorous approach offers the major advantage of isolating the true clinical effectiveness of the tool without the confounding factor of non-adherence, it leaves the question of organic compliance unaddressed—a factor that represents a significant component of a safety tool’s long-term viability in daily routine.

Furthermore, the lack of a prospective public protocol registration must be noted, alongside the fact that patient-specific systemic factors, such as smoking status, diabetes control, or exact ASA scores, were not systematically recorded in the initial protocol, limiting a deeper analysis of patient-related risk factors. Additionally, while our a priori power analysis was based on an expected reduction in workflow disruptions, the study ultimately demonstrated that the checklist primarily mitigated true clinical deviations. This suggests that the initial planning assumptions may have underestimated the inherent stability of organizational workflows in this specific setting. Finally, this protocol did not assess long-term implant success or survival rates, as clinical follow-up is inherently challenging in specialized surgical centers where implant placement and subsequent prosthetic rehabilitation are performed by different practitioners.

## Conclusion

In summary, this study demonstrates that implementing a standardized surgical checklist in implant dentistry provides organizational and clinical benefits. Despite generally low incident rates, we observed a reduction in clinical deviations and a significant decrease in procedure duration. These findings highlight that, when applied consistently, checklists improve overall team efficiency. Given the elective nature of implant surgery and the long-term impact of intraoperative precision, structured checklists should be regarded as a valuable and feasible addition to established surgical standards. To firmly establish this safety culture within the profession, its importance and use should be integrated into dental curricula and clinical training from an early stage.

## Supplementary Information

Below is the link to the electronic supplementary material.

Supplementary Material 1 (PDF 165 KB)

Supplementary Material 2 (PDF 63.0 KB)

## Data Availability

The datasets used and/or analyzed during the current study are available from the corresponding author on reasonable request.
