# Supplementary material for: Safer and faster: evaluation of a dental implant checklist
Source: Clin Oral Investig. 2026 Jun 29;30(7):304. doi: 10.1007/s00784-026-06986-6 (PMC13315161; doi:10.1007/s00784-026-06986-6)
Supplement: Supplementary file 2 — Supplementary Material 2 (PDF 63.0 KB) [file 784_2026_6986_MOESM2_ESM.pdf]

Implant
Checklist
Evaluation

# Data Collection Form

Age of the patient (Jahre):

Sex (f/m/d):

Surgery duration (minutes):

Number of inserted implants:

Bone augmentation (free-text):

| | ✓ | ✗ |
|---|---|---|
| Informed consent was obtained at least 24 hours before surgery. | ☐ | ☐ |
| The implant was placed in the correct patient. | ☐ | ☐ |
| The implant was placed in the correct position. | ☐ | ☐ |
| The patient experienced an allergic reaction. | ☐ | ☐ |
| The patient's blood glucose levels were outside the normal range. (Normal blood glucose levels assumed for non-diabetic patients.) | ☐ | ☐ |
| Unexpectedly severe bleeding occurred intraoperatively. | ☐ | ☐ |
| The patient experienced severe pain during treatment. | ☐ | ☐ |
| Nerve damage occurred. | ☐ | ☐ |
| Adjacent teeth were damaged. | ☐ | ☐ |
| Process disruptions occurred due to: | | |
| -missing or faulty material/equipment | ☐ | ☐ |
| -missing documentation | ☐ | ☐ |
| -missing radiographic images | ☐ | ☐ |
| -non-adherence to preoperative medication | ☐ | ☐ |
| Other incidents occurred (Free-text): | | |
| Assistance from another surgeon was required. | ☐ | ☐ |
